# Gut microbiota composition can reflect immune responses of latent tuberculosis infection in patients with poorly controlled diabetes

**DOI:** 10.1186/s12931-023-02312-w

**Published:** 2023-01-11

**Authors:** Hung-Ling Huang, Yong-Chun Luo, Po-Liang Lu, Cheng-Hsieh Huang, Kun-Der Lin, Meng-Rui Lee, Meng-Hsuan Cheng, Yao-Tsung Yeh, Cheng-Yuan Kao, Jann-Yuan Wang, Jinn-Moon Yang, Inn-Wen Chong

**Affiliations:** 1 Division of Pulmonary and Critical Care Medicine, Kaohsiung Medical University Hospital, 100, Shih-Chuan 1st Road, Kaohsiung, 80708 Taiwan; 2 Department of Internal Medicine, Kaohsiung Medical University Hospital, 100, Shih-Chuan 1st Road, Kaohsiung, 80708 Taiwan; 3 Department of Internal Medicine, Kaohsiung Municipal Ta-Tung Hospital, 68, Jhonghua 3rd Rd, Cianjin District, Kaohsiung, 80145 Taiwan; 4 Graduate Institute of Medicine, College of Medicine, Kaohsiung Medical University, 100, Shih-Chuan 1st Road, Kaohsiung, 80708 Taiwan; 5 Institute of Bioinformatics and Systems Biology, National Yang Ming Chiao Tung University, 1001, University Road Hsinchu, 30010 Taiwan; 6 Center for Liquid Biopsy and Cohort, Kaohsiung Medical University, 100, Shih-Chuan 1st Road, Kaohsiung, 80708 Taiwan; 7 Aging and Disease Prevention Research Center, Fooyin University, 151, Jinxue Rd., Daliao Dist., Kaohsiung, 83102 Taiwan; 8 Ph. D. Program in Environmental and Occupational Medicine, Kaohsiung Medical University, 100, Shih-Chuan 1st Road, Kaohsiung, 80708 Taiwan; 9 Department of Medical Laboratory Science and Biotechnology, Fooyin University, 151, Jinxue Rd., Daliao Dist., Kaohsiung, 83102 Taiwan; 10 Division of Endocrinology and Metabolism, Kaohsiung Medical University Hospital, 100, Shih-Chuan 1st Road, Kaohsiung, 80708 Taiwan; 11 Department of Internal Medicine, National Taiwan University Hospital, 7, Chung-Shan South Rd., Zhongzheng Dist., Taipei, 100225 Taiwan; 12 Department of Respiratory Therapy, Kaohsiung Medical University Hospital, 100, Shih-Chuan 1st Road, Kaohsiung, 80708 Taiwan; 13 Immunology Research Center, National Health Research Institutes, 35, Keyan Road, Zhunan Town, Miaoli, 35053 Taiwan; 14 Department of Biological Science and Technology, National Yang Ming Chiao Tung University, 75, Boai Street, Hsinchu, 300193 Taiwan; 15 Center for Intelligent Drug Systems and Smart Bio-Devices, National Yang Ming Chiao Tung University, 75 Boai Street, Hsinchu, 300193 Taiwan

**Keywords:** Gut microbiota, Latent tuberculosis infection, Immunity, Diabetic mellitus

## Abstract

**Background:**

Diabetes mellitus (DM) is a major risk factor for tuberculosis (TB). Evidence has linked the DM-related dysbiosis of gut microbiota to modifiable host immunity to *Mycobacterium tuberculosis* infection. However, the crosslinks between gut microbiota composition and immunological effects on the development of latent TB infection (LTBI) in DM patients remain uncertain.

**Methods:**

We prospectively obtained stool, blood samples, and medical records from 130 patients with poorly-controlled DM (pDM), defined as ever having an HbA1c > 9.0% within previous 1 year. Among them, 43 had LTBI, as determined by QuantiFERON-TB Gold in-Tube assay. The differences in the taxonomic diversity of gut microbiota between LTBI and non-LTBI groups were investigated using 16S ribosomal RNA sequencing, and a predictive algorithm was established using a random forest model. Serum cytokine levels were measured to determine their correlations with gut microbiota.

**Results:**

Compared with non-LTBI group, the microbiota in LTBI group displayed a similar alpha-diversity but different beta-diversity, featuring decrease of *Prevotella_9, Streptococcus*, and *Actinomyces* and increase of *Bacteroides, Alistipes,* and *Blautia* at the genus level. The accuracy was 0.872 for the LTBI prediction model using the aforementioned 6 microbiome-based biomarkers. Compared with the non-LTBI group, the LTBI group had a significantly lower serum levels of IL-17F (p = 0.025) and TNF-α (p = 0.038), which were correlated with the abundance of the aforementioned 6 taxa.

**Conclusions:**

The study results suggest that gut microbiome composition maybe associated with host immunity relevant to TB status, and gut microbial signature might be helpful for the diagnosis of LTBI.

**Supplementary Information:**

The online version contains supplementary material available at 10.1186/s12931-023-02312-w.

## Introduction

With the acceleration of the global burden of type 2 diabetes mellitus (DM) among people older than 40 years, DM has become a major threat for tuberculosis (TB) control [1, 2] because it compromises host immunity and facilitates either primary infection by *Mycobacterium tuberculosis* (*Mtb*) or reactivation from latent TB infection (LTBI) [3]. According to estimations, individuals with DM had a 3- and 1.18-fold higher risks of having active TB [4] or LTBI [5], respectively. Furthermore, every 1% increment of glycosylated hemoglobin (HbA1c) level can result in a 1.13-fold higher risk of LTBI [6]. A Taiwanese study revealed that approximately 26.7% of patients with poorly controlled diabetes, defined as ever having an HbA1c level > 9.0% in the past year, had LTBI. The prevalence was even higher than that among TB close contacts (15%) [7].

The effects of DM on host immune responses are complicated and may differ based on different TB statuses. Studies have suggested that although DM may augment systemic inflammation in patients with active TB, it attenuates mycobacteria-induced immune responses in those with LTBI by diminishing CD4-positive lymphocytes [8] and decreasing circulating levels of pro-inflammatory cytokines and anti-inflammatory cytokines [9]. The exact mechanism of disrupted host defense in DM patients with LTBI remains unknown, and host immunity enhancement might be a future strategy for preventing TB reactivation.

Recent advances in microbiome research indicate that gut microbiome–immune interactions in hosts are systemic, dynamic, and context dependent [10]. The transition and imbalance of gut microbiota can be observed under different DM statuses, which subsequently alters host immunity and homeostasis [11]. Additionally, growing evidence highlights the bidirectional modulation of lung immune responses through the enhancement of the niche-specific functions of the gut microbiome and its metabolites [12, 13]. Relevant gut microbiota alternations were associated with the disruption of host responses against TB infection in a healthy population [14], indicating that microbiota features could be modified to provide a critical asset in host responses to *Mtb* infection and serve as biomarkers to differentiate between different stages of TB.

This prospective cohort study aimed to investigate whether gut microbiota signatures can be used to identify LTBI status and reveal whether gut microbiota composition is correlated with cytokine levels in patients with poorly controlled DM (pDM).

## Materials and methods

### Study population and sample collection

This prospective study recruited patients with pDM, defined as those aged ≥ 45 years with a maximum HbA1c level of > 9.0% within the previous year before enrollment, between October 2019 and December 2020. Each patient with pDM received LTBI screening by using QuantiFERON-TB Gold In-tube (Cellestis/Qiagen, Carnegie, Australia). This study excluded patients with culture-confirmed active TB, history of LTBI treatment, malignancy, HIV infection, pregnancycy, concomitant acute or chronic inflammatory disease, and who used systemic antibiotics, immunosuppressive agents, or probiotics within 3 months prior to enrolment. Patients’ baseline characteristics and clinical medical records were obtained. The study was approved by the Institutional Ethics Committees of Kaohsiung Medical University Hospital (IRB: KMUIRB-G(II)-20170033, KMUHIRB-G(I)-20190035). Each participant provided informed consent before enrollment.

Fresh fecal samples were obtained from the recruited participants at enrollment for microbiota analysis, and blood samples were simultaneously collected for cytokine measurements. The fecal samples were stored in collection tubes with a DNA stabilizer, and the plasma isolated from blood samples was frozen at − 80 °C until further processing.

### Fecal sample processing and sequencing

Bacterial genomic DNA was extracted from the fecal samples using the QIAmp fast DNA Stool Mini Kit (Qiagen, Germany) according to the manufacturer’s protocol, and DNA was quantified using NanoDrop 2000 (Thermo Fisher Scientific, USA).

DNA libraries were produced by polymerase chain reaction amplicons targeting the V3 and V4 hypervariable regions of the 16S rRNA gene by using 341F and 805R primers, and were sequenced using the Illumina MiSeq (Illumina, USA) 2 × 300-bp paired-end reads platform (details in Additional file 1).

### Bioinformatics analysis

Demultiplexed sequencing reads were trimmed using QIIME2.0 and filtered for quality using a DADA2 pipeline; subsequently, the reads were merged into amplicon sequence variants (ASVs) for downstream analysis. Taxonomy identification was mapped based on the SILVA 16S rRNA gene reference database (version 132) [15].

Alpha-diversity metrics, including observed ASVs, Faith’s phylogenetic diversity (PD), Shannon index, and Pielou’s evenness index were compared between groups using Mann–Whitney *U* tests with correction for multiple comparison using the Benjamini–Hochberg method [16]. For the beta-diversity analysis, UniFrac distances were used to illustrate microbial community structures, and permutational multivariate analysis of variance (PERMANOVA) was used to determine taxonomic differences between LTBI and non-LTBI groups. Principal coordinate analysis (PCoA) enabled the visualization of unweighted and weighted UniFrac distances [17]. Linear discriminant analysis (LDA) and LDA with effect size measurements (LEfSe) were used to evaluate the influence of each differentially abundant taxon between groups [18]. Pathway enrichment analysis was performed using the Kyoto Encyclopedia of Genes and Genomes (KEGG) and SILVA reference database by PICRUSt2 [19].

#### Plasma cytokine measurement

The serum concentrations (pg/mL) of interleukin (IL)-17A, IL-17F, IL-22, interferon gamma (IFN-γ), IL-2, IL-10, IL-22, and tumor necrosis factor-alpha (TNF-α) were measured using enzyme‐linked immunofluorescence assay (ELISA) kits according to the manufacturer’s protocol (R&D Systems Inc., Minneapolis, Minnesota, USA).

### Statistical analysis

Statistical analysis was performed using SPSS version 22 (IBM, Armonk, NY, USA). Continuous data were expressed as mean ± standard error of mean or standard deviation and compared using Student’s *t* test or Mann–Whitney *U* test based on the normality of data distribution. Categorical data were expressed as percentages and compared using chi-squared test. A linear regression model was applied to analyze the correlation between microbial relative abundance and cytokine level. Statistical significance was denoted by p < 0.05.

### Random forest model creation for predicting LTBI in patients with pDM

A random forest model was constructed using the RandomForest Classifier from the scikit-learn Python library. The study cohort was randomly divided into training and test sets (70:30). Each decision tree in the forest classified samples as being from participants with or without LTBI based on differential taxa abundance, and the model parameters included number of trees (1000), number of top genera (from 2 to 26), and other default parameters to retain consistency among comparisons; the parameters were systemically tested using bootstrapping to avoid overfitting. The feature importance score was computed based on Gini index [20] (details in Additional file 1). A confusion matrix was constructed to calculate the accuracy, sensitivity, specificity and F1 score to predict LTBI status based on the relative abundance of top genera. The areas under the receiver operating characteristic curve (AUROC) was used for evaluating the discriminative ability of the model.

## Results

### Characteristics of enrolled participants

**Fig. 1:**
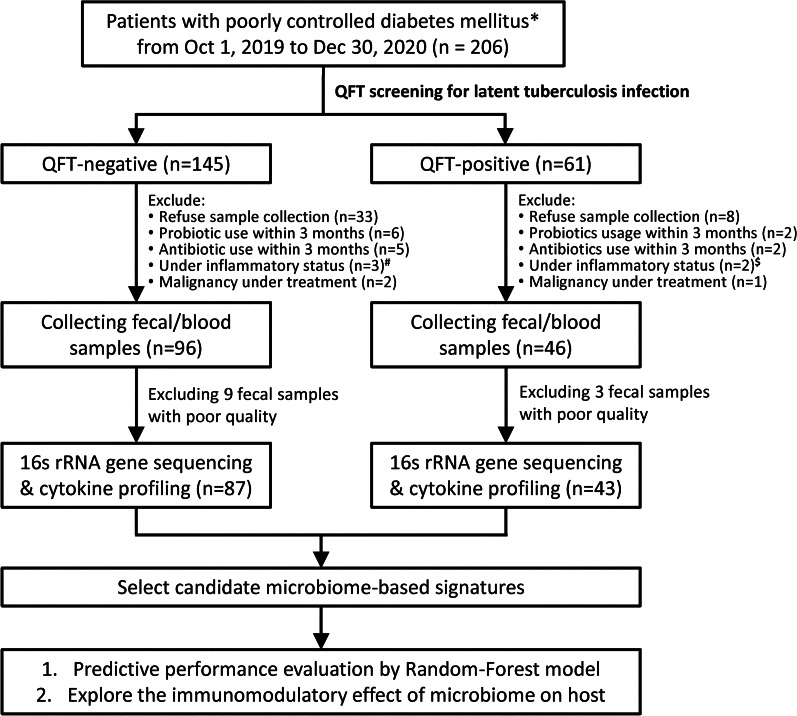
Overview of case selection, sample collection, and analysis strategies. QFT: QuantiFERON-TB Gold In-tube. * Poorly controlled diabetes mellitus was defined as ever having a glycated hemoglobin (HbA1c) level of ≥ 9.0% within 1 year before enrollment. ^#^ Two patients had bronchitis and one had urinary tract infection. ^$^ One patient had influenza A and one had urinary tract infection

Figure 1 presents the patient selection process. In total, 130 participants with pDM (LTBI = 43; non-LTBI = 87) were included in the final analysis.

Table 1 and Additional file 1: Table S1 present the clinical, laboratory, and immunological profiles of the 130 patients with pDM. Among them, 51% were male. The mean age was 67.1 years, mean body mass index (BMI) was 26.6 kg/m^2^, 80.0% were nonsmokers, and the mean maximum HbA1c level within 1 year was 10.7%. The baseline characteristics (age, sex, BMI, diet, and use of antihyperglycemic and lipid-lowering drugs) were similar between LTBI and non-LTBI groups. The mean HbA1c level at enrollment was 8.8% and 8.9% in the LTBI and non-LTBI groups, respectively (p = 0.723).

**Table 1:** Clinical characteristics of the 130 patients with poorly controlled diabetes mellitus, stratified by the status of latent tuberculosis infection (LTBI)

	All (n = 130)	Non-LTBI (n = 87)	LTBI (n = 43)	*p*-value
Male sex	66 (51%)	47 (54%)	19 (44%)	0.291
Age	67.1 ± 7.6	66.1 ± 9.8	68.2 ± 9.0	0.121
BMI (kg/m^2^)	26.6 ± 4.2	26.7 ± 4.5	26.6 ± 3.4	0.395
Smoking status				0.694
Never smoker	104 (80%)	68 (78%)	36 (84%)	
Ex-smoker	13 (10%)	9 (10%)	4 (9%)	
Current smoker	13 (10%)	10 (11%)	3 (7%)	
Dietary (times/day)				
Fruit/Vegetables	1.0 ± 0.6	1.0 ± 0.6	1.0 ± 0.7	0.916
Seafood	0.9 ± 0.5	0.9 ± 0.5	0.9 ± 0.5	0.976
Processed meat	1.1 ± 0.6	1.2 ± 0.6	1.1 ± 0.6	0.858
Comorbidities				
Hypertension	93 (72%)	58 (67%)	35 (81%)	0.080
Old CVA	13 (10%)	9 (10%)	4 (9%)	0.885
CKD, stage ≥ 3	45 (35%)	32 (37%)	13 (30%)	0.460
ESRD	2 (2%)	2 (2%)	0 (0%)	> 0.999
Coronary artery disease	15 (12%)	8 (9%)	7 (17%)	0.215
Hepatitis B	5 (4%)	3 (3%)	2 (5%)	0.737
Hepatitis C	5 (4%)	2 (2%)	3 (7%)	0.331
Laboratory data				
Maximum HbA1c (%)	10.7 ± 1.7	10.8 ± 1.8	10.5 ± 1.5	0.430
HbA1c at enrolment (%)	8.8 ± 1.7	8.9 ± 1.8	8.8 ± 1.4	0.723
Total cholesterol (mg/dL)	181.4 ± 40.2	177.9 ± 39.0	189.4 ± 41.4	0.122
LDL (mg/dL)	98.9 ± 34.5	96.3 ± 31.0	106.9 ± 40.5	0.101
HDL (mg/dL)	47.7 ± 28.5	47.8 ± 32.6	44.5 ± 13.4	0.529
Triglyceride (mg/dL)	173.5 ± 127.4	157.6 ± 114.5	202.0 ± 143.5	0.081
Antihyperglycemic drug				
Insulin	80 (62%)	50 (57%)	30 (70%)	0.175
Metformin	96 (74%)	64 (74%)	32 (74%)	0.917
Sulfonylurea	69 (53%)	50 (57%)	19 (44%)	0.153
DPP-4 inhibitor	66 (51%)	44 (51%)	22 (51%)	0.950
SGLT2 inhibitor	46 (35%)	31 (36%)	15 (35%)	0.933
Thiazolidinedione	47 (36%)	35 (40%)	12 (28%)	0.169
Meglitinide	12 (9%)	8 (9%)	4 (9%)	0.984
α-glucosidase inhibitor	12 (9%)	9 (10%)	3 (7%)	0.533
GLP-1 agonist	6 (5%)	3 (3%)	3 (7%)	0.397
Lipid-lowering drug				
Statin	84 (65%)	53 (61%)	31 (72%)	0.210
Fibrate	23 (17%)	14 (16%)	9 (21%)	0.496

CKD: chronic kidney disease; CAD: coronary artery disease; CVA: cerebral vascular accident; DM: diabetic mellitus; DPP-4: dipeptidyl peptidase-4; ESRD: end-stage renal disease; GLP-1: glucagon-like peptide 1; HbA1c: glycated hemoglobin; HDL: high density lipoprotein; LDL: low density lipoprotein; LTBI: latent tuberculosis infection; SGLT2: sodium glucose co-transporters 2

Data are either mean ± standard deviation or number (%)

*p* value was calculated using χ^2^ test, student’s *t* test and Mann–Whitney *U* test, if appropriate

### Sequencing data and microbiome characteristics

**Fig. 2:**
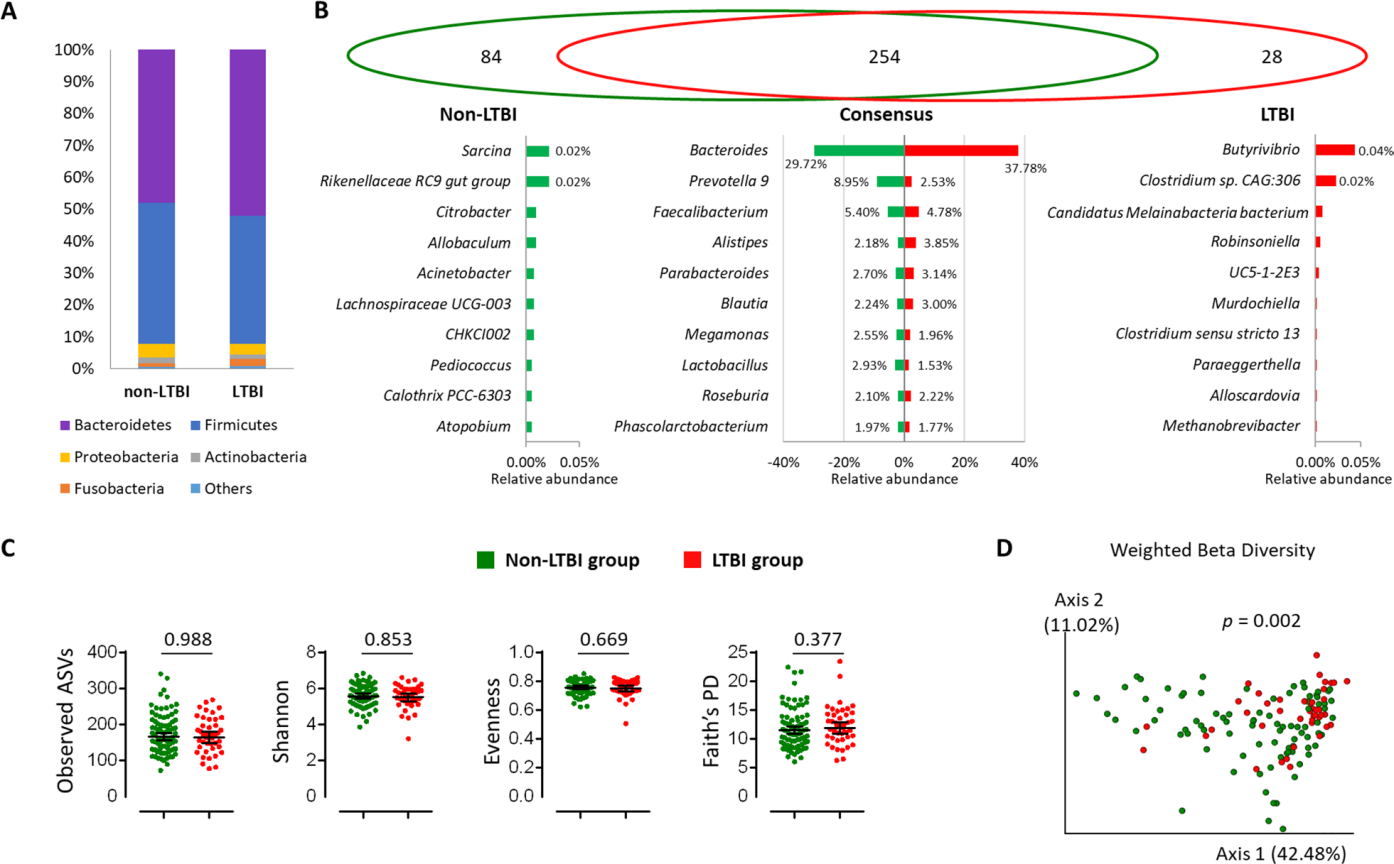
Altered biodiversity and the major components of gut microbial communities in diabetes mellitus (DM) patients with or without latent tuberculosis infection (LTBI). **A** The top 10 phyla in LTBI and non-LTBI groups are shown with their abundance. **B** The top 10 genera present in both LTBI and non-LTBI groups (254 genera; center panel), only in the non-LTBI group (84 genera; left panel), and only in the LTBI group (28 genera; right panel) are presented with their abundance. **C** The alpha-diversity, namely the richness of gut microbes, was determined by the observed amplicon sequence variants (ASVs), Shannon index, Pielou’s evenness index, and Faith’s phylogenetic diversity (PD). **D** Beta-diversity was determined by a principal coordinates analysis (PCoA) plot based on the weighted UniFrac distance. Each dot represents one sample from each group. The relevant *p* values were calculated using a Mann–Whitney *U* test

In total, 16,147,438 16S rRNA reads were generated from fecal samples provided by the 130 participants, and 6,813,529 reads and 4371 ASVs were obtained after denoising and filtering were conducted. We obtained a median of 49,423 sequences (range: 32,136–121,226) per sample and used 45,000 sequences per sample for rarefaction, revealing that saturation was reached (see Additional file 1: Fig. S1). Following taxonomic assignment, all ASVs were aligned to 366 genera and 871 species. Figure 2A/2B and Additional file 1: Tables S2–S5 reveal the relatively abundant composition of gut microbiota at phylum and genus level in LTBI and non-LTBI groups. *Bacteroides* was more abundant in the LTBI group (37.79% vs. 29.72%, p = 0.001), whereas *Prevotella_9* was more abundant in the non-LTBI group (2.53% vs. 8.95%, p < 0.001).

### Biodiversity of gut microbiota is associated with LTBI status in patients with pDM

Figure 2C indicates that no differences exist between LTBI and non-LTBI groups in terms of alpha-diversity based on observed ASVs (p = 0.988), Shannon index (p = 0.853), evenness (p = 0.669), and Faith’s PD (p = 0.377). By contrast, beta-diversity was significantly different between the 2 groups, as determined using PCoA plots based on the unweighted and weighted UniFrac distance (p = 0.007 and p = 0.002 by PERMANOVA, respectively) (Fig. 2D and Additional file 1: Fig. S2A). The LTBI group had a significantly lower *Prevotella*/*Bacteroides* ratio than non-LTBI group did (0.251 vs. 0.724, p < 0.001, Additional file 1: Fig. S2B). Additionally, there was no differences in beta-diversity among subgroups stratified based on different clinical characteristics, including sex, obesity (body-mass-index ≥ 27 kg/m^2^), use of various antidiabetic and antihyperlipidemic drugs (Additional file 1: Fig. S3).

**Fig. 3:**
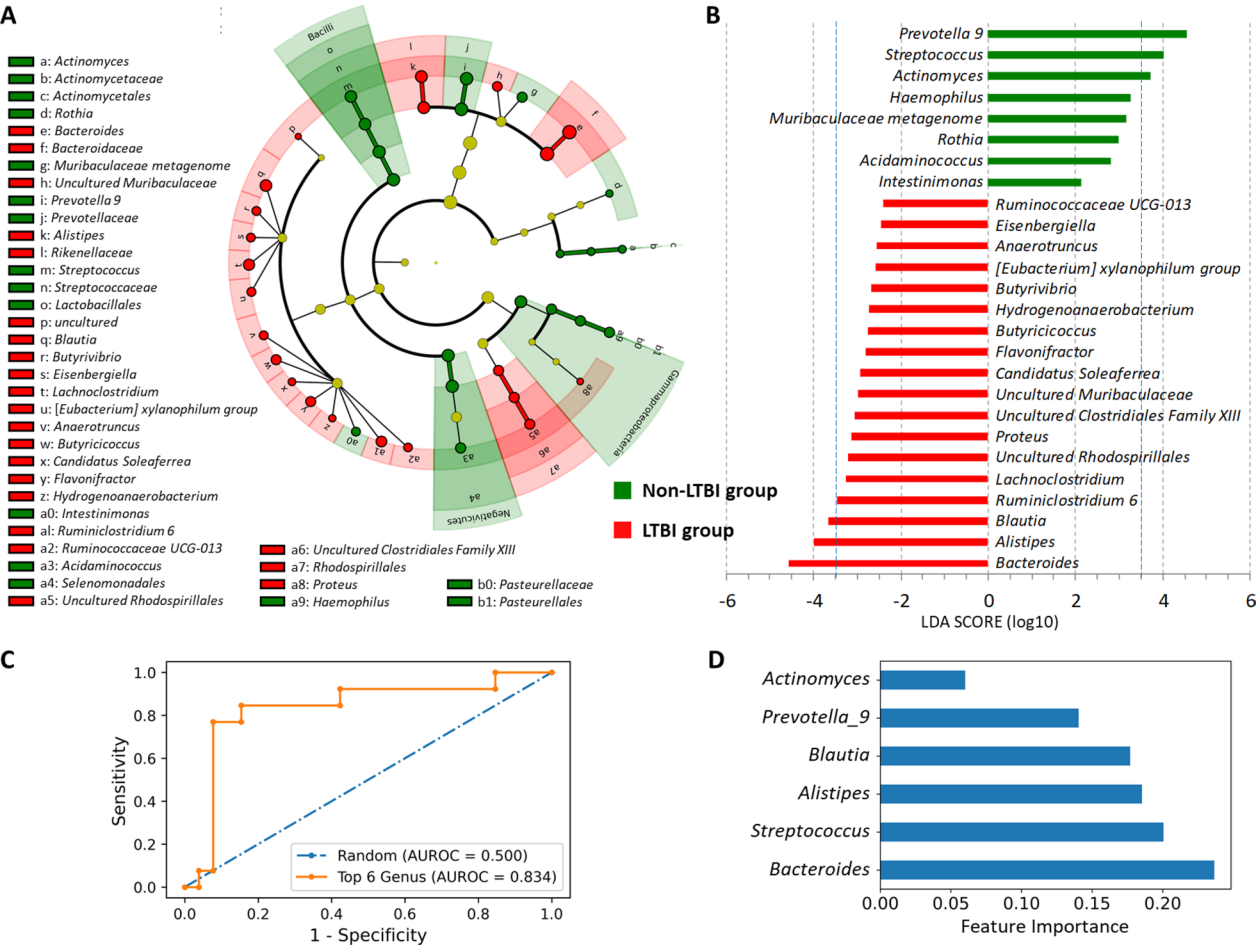
Differential abundance analysis and identification of representative genera as predictive signatures through linear discriminant analysis (LDA) with effect size measurements (LEfSe) analysis and a random forest model to discriminate between patients with poorly controlled diabetes with and without latent tuberculosis infection (LTBI). **A** Significantly different taxa in the cladogram according to a LDA score of ≥ 2 (each circle represents phylogenetic levels from phylum to genus [inside to outside], and each diameter is proportional to the taxon’s abundance). **B** Significantly different genera in terms of relative abundance (LDA score of ≥ 2) between LTBI and non-LTBI groups. **C** Receiver operating characteristics (ROC) curve analysis of the predictive model using the 6 most differentially abundant genera (*Bacteroides, Alistipes*, *Blautia*, *Prevotella_9, Streptococcus,* and *Actinomyces*) for discriminating between LTBI and non-LTBI groups (AUROC: area under the ROC curve). **D** Feature importance of each of the 6 genera in the predictive model

LEfSe revealed 41 discriminating taxon features between LTBI and non-LTBI groups across different phylogenetic levels, including 3 classes, 5 orders, 7 families, and 26 genera (LDA score (log10) > 2.0, p < 0.05; Fig. 3A and B and Additional file 1: Table S6). Compositional differences in gut microbiota at the genus level (LDA score (log10) > 3.5) were primarily driven by the enrichment of *Bacteroides*, *Alistipes* and *Blautia* in the LTBI group and the enrichment of *Prevotella_9*, *Streptococcus*, and *Actinomyces* in the non-LTBI group.

### Gut microbiota-based prediction for discriminating between patients with and without LTBI

In accordance with the 6 most differential genera between the LTBI and non-LTBI groups for predicting LTBI status in the 130 patients with pDM, the best random forest model constructed had an AUROC of 0.834, an accuracy of 0.872, sensitivity of 0.769, and specificity of 0.923 (Fig. 3C and Additional file 1: Tables S7–S8). Regarding feature importance in this predictive model, *Bacteroides* was the genus with the highest weighting, followed by *Streptococcus, Alistipes, Blautia, Prevotella_9,* and *Actinomyces* (Fig. 3D)*.*

### Plasma cytokine level is correlated with gut microbiota composition

**Fig. 4:**
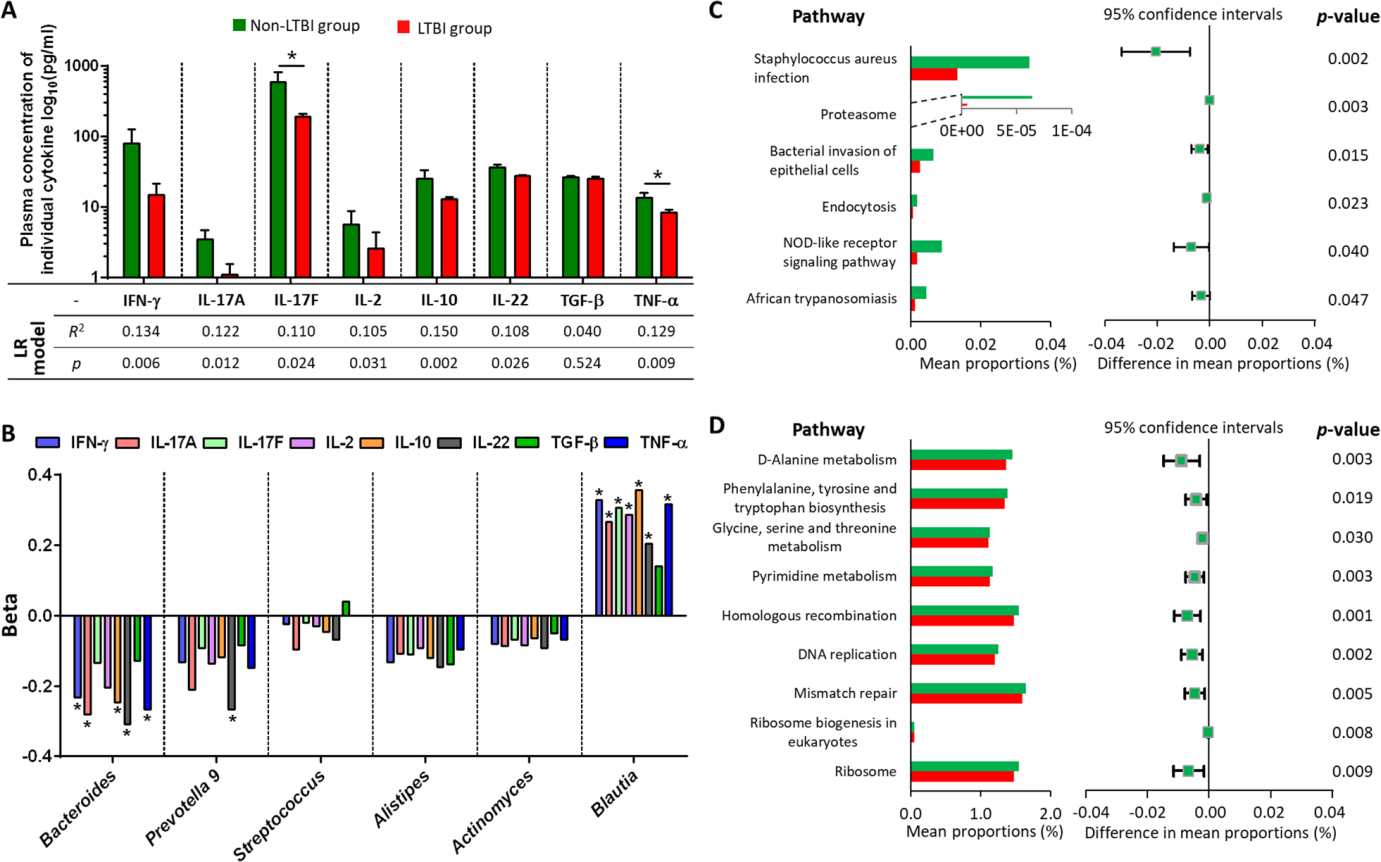
Correlation of the plasma levels of individual cytokines with latent tuberculosis infection (LTBI) status and the 6 most differentially abundant genera (*Bacteroides, Alistipes*, *Blautia*, *Prevotella_9, Streptococcus,* and *Actinomyces*) (* represents *p* < 0.05). **A** Plasma cytokine levels (mean ± standard error mean, pg/mL) between LTBI and non-LTBI groups (compared using a Mann–Whitney *U* test) and the overall performance of the linear regression (LR) models in predicting the individual levels of plasma cytokines. **B** Beta estimates related to the 6 selected genera in the 6 LR models for predicting individual cytokine levels among overall population. **C** Gene functions related to immune, **D** metabolism, and replication pathways in the gut microbiota according to PICRUSt2 with an extended error bar plot to demonstrate the difference between LTBI and non-LTBI groups (the middle value represents the mean inter-group differences with the non-LTBI group used as a reference, and the error bar represents the relevant 95% confidence interval)

Compared with non-LTBI group, the plasma concentrations of IL-17F and TNF-α, which indicate cytokine responses from T helper-17 (Th17) cells and T helper-1 (Th1) cells, respectively, were significantly lower in the LTBI groups (p = 0.025 and p = 0.038, respectively; Fig. 4A and Additional file 1: Table S1). The 11 potential confounders, including sex, obesity (BMI ≥ 27 kg/m^2^), use of various antidiabetic medications, and statin, were poorly correlated with plasma cytokine levels of IFN-γ, IL-17A, IL-17F, IL-2, IL-10, IL-22, TNF-α and TGF-β, and the model performance improved after adding the 6 selected genera into the linear regression model (Additional file 1: Tables S9 and S10). Additionally, the 6 gut microbiota signatures accounted for the majority of top 5 important features in linear regression model (Additional file 1: Fig. S4).

Regarding the effect of individual genera, *Blautia* was positively correlated with all measured cytokines except for TGF-β; *Bacteroides* had a negative correlation with IFN-γ, IL-17A, IL-10, IL-22, and TNF-α; and *Prevotella 9* was negatively associated with IL-22 (Fig. 4B and Additional file 1: Table S9).

### Functional analysis

In total, 20 and 43 differentially abundant pathways were significantly modified at KEGG levels 2 and 3, respectively (p < 0.05), suggesting diverse changes in the functions of microbiota between LTBI and non-LTBI groups (Additional file 1: Fig. S5). Most of the predicted microbial functions in patients with LTBI are negatively correlated with immune (Fig. 4C), metabolism, and replication (Fig. 4D) pathways**.**

## Discussion

This is the first study to unravel links between LTBI status, host immunity, and gut microbiome dysbiosis in patients with pDM. We demonstrated the following: (1) pDM patients with LTBI have a markedly different gut microbiome to individuals without LTBI; they had enriched *Bacteroides, Alistipes*, and *Blautia*, but depleted *Prevotella_9*, *Streptococcus*, and *Actinomyces*. (2) The proposed microbiota-based model involving the 6 most differential genera in gut microbiota had favorable performance with an accuracy of 0.872 in predicting LTBI status among patients with pDM. (3) The 6 discriminatory genera were correlated with a decrease in Th1/Th17 cell-mediated inflammatory cytokines and indicated to involve in depletion of immune, metabolism, and replication pathways.

Interferon gamma release assays (IGRAs), based on the detection of IFN-γ responses of peripheral lymphocytes against *Mtb*-specific antigens, are the current standard diagnostic test for LTBI. However, they have a low sensitivity in patients with DM, most likely because of the attenuation of IFN-γ release [21, 22]. Additionally, the results of sequential IGRAs cannot reflect dynamic changes in *Mtb* infection and identify subclinical TB [23]. A new diagnostic platform incorporating various diagnostic modalities for LTBI is necessary to guide point-of-care management and the timing of treatment initiation.

Studies have demonstrated that compared with healthy individuals, the gut microbiota of patients with DM have a lower relative abundance of genera *Bacteroides* [24], *Alistepes* [25] and *Blautia* [26] and a higher relative abundance of *Actinomyces* [24] and *Prevotella* [27]*.* We however demonstrated that the DM patients with LTBI had higher abundance of *Bacteroides, Alistipes*, and *Blautia* than those without LTBI, implying that LTBI status might be associated with the dysbiotic microbiota of patients with pDM.

Furthermore, growing evidence indicates that the disruption of gut microbiome equilibrium can contribute to changes in TB stage [14, 28, 29], which could provide clinical utility for LTBI diagnosis beyond conventional IGRAs.

The most differential bacterial taxa between pDM patients with and without LTBI identified by the current prediction model have previously been associated with TB susceptibility based on the regulation of Th1/Th17 immune responses and inflammation [14, 28–31]. Although the role of *Streptococcus* and *Actinomyces* had not previously been identified in patients with TB, patients with active TB have a lower phylogenetic diversity and a significantly lower abundance of short chain fatty acid (SCFA)–producing bacteria such as *Bacteroidetes*, *Alistepes*, and *Prevotella* compared with healthy individuals [32–34]. Conversely, the unique gut microbiome features with high abundance of SCFA producers (*Bacteroides* and *Alistepes*) in current pDM patients with LTBI may enhance TB susceptibility by suppressing B cells and CD4+ and CD8+ lymphocytes, reducing the production of TB-induced IFN-γ and IL-17, increasing *Foxp1* expression [14, 28, 30, 31] and elevating the number of T regulatory cells in peripheral blood [29]. *Blautia* was reported to be more abundant in patients with TB than in symptomatic patients without TB and transcriptome analysis indicated that this abundance may be related to inflammation-modulating pathways [35]. Paradoxically, *Prevotella* had diverse immunomodulatory properties in different TB stages; it was positively correlated with CD4+ cell counts in patients with newly diagnosed TB but negatively correlation with such cell counts in patients with TB recurrence [33]. Further mechanistic studies are required to confirm the immunomodulatory effect of each relevant taxon.

In accordance with the findings in previous studies [9, 36], this study revealed significantly lower plasma levels of TNF-α and IL-17 in DM patients with LTBI than non-LTBI counterparts, suggesting increased TB susceptibility through the decreasing phagocytic ability of macrophages, interference with granuloma formation [37] and inhibiting *Mtb*-specific memory responses [38]. We though herein demonstrated the significant correlation between Th1- and Th17-related cytokines and the 6 most differentially abundant taxa of gut microbiota, further studies should be conducted to explore the immunomodulatory effect of gut microbiome on *Mtb* infection.

Furthermore, the significant downregulation of gene expression in immune, metabolism, and replication pathways in the LTBI group was in accordance with the evasion of immune surveillance through the suppression of host immunity, a reduction in energy expenditure, and the attainment of an intracellularly nonreplicant dormancy status by *Mtb* [39]. Taken together, the findings of the current study provide preliminary evidence that gut microbiota composition may reflect the immune response of pDM patients with different status of *Mtb* infection. Furthermore, the findings could provide a first step toward host-directed immunomodulatory therapy through the precise tuning of the enteric microbiome to enhance host immunity against *Mtb* infection.

This study had several limitations. First, this cross-sectional study could not determine causality or the mechanisms behind the effect of gut microbiota alterations on host immunity during various stages of *Mtb* infection. Second, using 16s rRNA sequencing rather than shotgun metagenomics may interfere the taxonomic and functional resolution of microbiomes owing to the inadequacy of gene-related information obtained from strains. Third, current study did not reveal the influence of microbiome-derived metabolite alterations on TB pathogenesis. Further metabolomics studies should be conducted to verify this influence. Forth, ethnicity and regional variations among individuals can alter the precision of microbiome-based diagnostics. An external validation of the proposed prediction model is necessary.

### Conclusion

Our study indicates that gut microbiome composition could modulate Th1/Th17 cell–mediated immune responses, which are potentially relevant to TB susceptibility among patients with pDM. We provide a gut microbiome-based prediction model for discriminating between pDM patients with and without LTBI status. This study provides a foundation for explorations relevant to gut microbiome-based diagnostic biomarkers and host-directed treatment strategies; such explorations could alleviate the TB–DM co-epidemic.

## Supplementary Information

**Additional file 1: Table S1.** Laboratory and cytokine analysis results for the 130 patients with poorly controlled diabetes mellitus. **Table S2.** Relative abundance (%) of the top 10 phyla in both the latent tuberculosis infection (LTBI) group and non-LTBI groups. **Table S3.** Relative abundance (%) of the top 10 genera in both latent tuberculosis infection (LTBI) group and non-LTBI group. **Table S4.** Relative abundance (%) of the top 10 genera only in the non-latent tuberculosis infection (non-LTBI) group. **Table S5.** Relative abundance (%) of the top 10 genera only in the latent tuberculosis infection (LTBI) group. **Table S6.** Relative abundance (%) of the 26 most differential genera between the latent tuberculosis infection (LTBI) and non-LTBI groups. **Table S7.** Performance of predictive models that included different numbers of the 26 most differential genera between the latent tuberculosis infection (LTBI) and non-LTBI groups. **Table S8.** Confusion matrix of the classifier involving 6 genera and a test set (39 samples [30%]) for differentiating between latent tuberculosis infection (LTBI) and non-LTBI groups, as determined by a random forest model. **Table S9.** P values for each of the 6 selected genera in linear regression models for predicting the plasma levels of individual cytokines and the proposed model performance. **Table S10.** Change of model performance for predicting the plasma levels of individual cytokines before and after including the 6 selected genera into the linear regression models containing 11 potential confounders (sex, body-mass index ≥ 27 kg/m^2^, and use of metformin, DDP4 inhibitor, SGLT2 inhibitor, sulfonylurea, thiazolidinedione, meglitinides, acarbose, and use of ≥ 3 oral antidiabetic drugs, as well as statin). **Figure S1.** Rarefaction curve of sequencing data from 130 fecal samples. Samples with fewer than 45,000 sequences were excluded, and the remaining samples were rarefied to 49,423 sequences (range: 32,136 to 121,226) per sample for subsequent ordinations and permutational multivariate ANOVA analysis.Abbreviations: ASVs, amplicon sequence variants. **Figure S2.** Biodiversity and Prevotella/Bacteroides (P/B) ratio of gut microbial communities in diabetes mellitus (DM) patients with or without latent tuberculosis infection (LTBI). (*A*) Beta diversity was determined by a principal coordinates analysis plot based on the unweighted UniFrac distance. Each dot represents one sample. (**B**) Prevotella/Bacteroides (P/B) ratio between the latent tuberculosis infection (LTBI) and non-LTBI groups. *** represents a *P* value of < 0.001 according to a Mann–Whitney *U* test. **Figure S3.** Biodiversity of gut microbial communities in diabetes mellitus (DM) patients with or without potential confounders: (**A**) sex, (**B**) BMI ≥ 27kg/m2, (**C**) Metformin, (**D**) DDP4 inhibitor, (**E**) SGLT2 inhibitor, (**F**) Sulfonylurea, (**G**) Thiazolidinedione, (**H**) Meglitinide, (**I**) Acarbose, (**J**) Statin. **Figure S4.** The feature importance of the 6 selected genera and potential confounders in the predicting models for serum cytokine levels. **Figure S5.** Prediction of gene function of the 6 most differential genera between latent tuberculosis infection (LTBI) and non-LTBI groups using PICRUSt2.

## Data Availability

Sequencing data for all samples used in this study have been deposited in https://www.ncbi.nlm.nih.gov/geo/query/acc.cgi?acc=GSE199810. Deidentified individual participant data that underlie the results reported in this article will be available immediately following publication upon request to the corresponding author.

## Abbreviations

ASVs: Amplicon sequence variants
AUROC: Areas under the receiver operating characteristic curve
DM: Diabetes mellitus
HbA1c: Glycosylated hemoglobin
IL: Interleukin
IFN-γ: Interferon gamma
LTBI: Latent TB infection
KEGG: Kyoto Encyclopedia of Genes and Genomes
LDA: Linear discriminant analysis
LEfSe: LDA with effect size measurements
PCoA: Principal coordinate analysis
PD: Phylogenetic diversity
pDM: Poorly controlled diabetes
PERMANOVA: Permutational multivariate analysis of variance
RF model: Random forest model
TB: Tuberculosis
TNF-α: Tumor necrosis factor-alpha
