# Supplementary material for: Gut microbiota composition can reflect immune responses of latent tuberculosis infection in patients with poorly controlled diabetes
Source: Respir Res. 2023 Jan 11;24:11. doi: 10.1186/s12931-023-02312-w (PMC9835344; doi:10.1186/s12931-023-02312-w)

**Additional file 1**

**Title:** Gut microbiota composition can reflect immune responses of latent tuberculosis infection in patients with poorly controlled diabetes

**Authors:** Hung-Ling Huang,^1,2,3,4^, Yong-Chun Luo,^5^ Po-Liang Lu,^2,4,6^ Cheng-Hsieh Huang,^7,8,9^ Kun-Der Lin,^2,4,10^ Meng-Rui Lee,^11^ Meng-Hsuan Cheng,^1,2,4,12^ Yao-Tsung Yeh,^9^ Cheng-Yuan Kao^13^, Jann-Yuan Wang,^11,^**^§^** Jinn-Moon Yang,^5,14,15,^**^§^** Inn-Wen Chong,^1,2,4,14,^ **^§^**

**Affiliations:**

^1^Division of Pulmonary and Critical Care Medicine, ^2^Department of Internal Medicine, ^10^Division of Endocrinology and Metabolism,^12^Department of Respiratory Therapy, Kaohsiung Medical University Hospital, Kaohsiung, Taiwan.

^3^Department of Internal Medicine, Kaohsiung Municipal Ta-Tung Hospital, Kaohsiung, Taiwan

^4^Graduate Institute of Medicine, College of Medicine, ^8^Ph. D. Program in Environmental and Occupational Medicine,^6^Center for Liquid Biopsy and Cohort, Kaohsiung Medical University, Kaohsiung, Taiwan

^5^Institute of Bioinformatics and Systems Biology, ^14^Department of Biological Science and Technology, ^15^Center for Intelligent Drug Systems and Smart Bio-devices, National Yang Ming Chiao Tung University, Hsinchu, Taiwan

^7^Aging and Disease Prevention Research Center, ^9^Department of Medical Laboratory Science and Biotechnology, Fooyin University, Kaohsiung, Taiwan.

^11^Department of Internal Medicine, National Taiwan University Hospital, Taipei, Taiwan

^13^Immunology Research Center, National Health Research Institutes, Miaoli County, Taiwan

**^§^** The three authors contributed equally

**Corresponding author**

Dr. Jann-Yuan Wang

Department of Internal Medicine, National Taiwan University Hospital

#7, Chung-Shan South Rd., Zhongzheng Dist., Taipei 100225, Taiwan

Email: jywang@ntu.edu.tw

Tel: 886-2-23123456 ext 63565

Fax: 886-2-23582867

**MATERIALS AND METHODS**

**Fecal processing and sequencing**

1. Fecal DNA extraction: Bacterial genomic DNA was extracted from fecal samples using the QIAmp fast DNA Stool Mini Kit (Qiagen, Germany) based on modified instructions. In brief, stool samples were centrifugated at 10,000 rpm for 3 min to remove the storage buffer and lysed with InhibitEX Buffer, proteinase K, RNase A, and ethanol to obtain the processed supernatant. The supernatant was then washed using a QIAamp spin column and eluted with a preheated elution buffer. The concentration was assessed using NanoDrop 2000.
2. Sequencing analysis: The sequencing library was constructed based on the standard V3–V4 region of the 16S rRNA gene by using 341F and 805R primers (5-TCGTCGGCAGCGTCAGATGTGTATAAGAGACAGCCTACGGGNGGCWGCAG and 5-GTCTCGTGGGCTCGGAGATGTGTATAAGAGACAGGACTACHVGGGTATCTAATCC, respectively). Polymerase chain reaction (PCR) products were amplified using the KAPA HiFi hotstart readymix (Roche, USA) and purified using AMPure XP magnetic beads (Beckman Coulter, USA). The quality of PCR products was assessed using a Fragment Analyzer (Advanced Analytical, USA), and their quantity was determined using a Qubit 3.0 Fluorometer. The library was then sequenced using MiSeq (Illumina, USA) with paired-end reads (2 × 300 nt) and at least 100,000 reads of every sample.

**Feature importance in a random forest model**

The feature importance score was computed to measure the degree of association between the classification results and a given feature based on the Gini index [1]. The Gini index *i*(*S*) of node *S* is calculated as follows:

| $i\left( S \right)=1-\sum_{i=1}^{k} p_{i}^{2}$ | (1) |
| --- | --- |

where *k* is the number of classes, *p_i_* is the probability calculated as *n_i_*/*n*, *n* is the total samples in node *S*, and *n_i_* is number of samples in class *i*. *i*(*S*) decreases when the node *S* is split to 2 child nodes: the left node (*S_l_*) and right node (*S_r_*). The decrease in the Gini index of node *S*, Δ*i*(*S*), is calculated as follows:

| $\Delta i\left( S \right)=i\left( S \right)-p_{l}\times i\left( S_{l} \right)-p_{r}\times i(S_{r})$ | (2) |
| --- | --- |

where *p_l_* and *p_r_* are the probability calculated as *n_l_*/*n* and *n_r_*/*n*, respectively. *i*(*S_l_*) and *i*(*S_r_*) are the Gini index of the left child node and right child node split by node *S*, respectively. For each feature, the average Δ*i*(*S*) is computed on each tree and normalize to a value between 0 and 1, and the final importance score is the sum of the normalized score divide by the total number of trees.

**Reference:**

1. Menze BH, Kelm BM, Masuch R, et al. A comparison of random forest and its Gini importance with standard chemometric methods for the feature selection and classification of spectral data. BMC Bioinform **2009**; 10: 213.

**Additional file 1: Table S1**. Laboratory and cytokine analysis results for the 130 patients with poorly controlled diabetes mellitus

|  | **All**  **(n = 130)** | **Non-LTBI**  **(n = 87)** | **LTBI**  **(n = 43)** | ***P***  **value** |
| --- | --- | --- | --- | --- |
| Laboratory data |  |  |  |  |
| Leukocyte (x 1000/uL) | 7.2 ± 1.7 | 7.3 ± 1.7 | 7.1 ± 1.8 | 0.557 |
| Hemoglobin (g/dL) | 13.4 ± 2.0 | 13.4 ± 2.0 | 13.6 ± 2.0 | 0.487 |
| Platelet (x 1000/uL) | 241 ± 51 | 244 ± 52 | 235 ± 48 | 0.294 |
| AST (U/L) | 28.4 ± 19.9 | 30.1 ± 22.3 | 27.3 ± 16.9 | 0.216 |
| ALT (U/L) | 26.3 ± 13.7 | 26.9 ± 15.3 | 25.0 ± 10.0 | 0.454 |
| Creatinine (mg/dL) | 1.2 ± 1.1 | 1.2 ± 1.1 | 1.2 ± 0.9 | 0.912 |
| QFT test (IU/mL) |  |  |  |  |
| Nil |  | 0.1 ± 0.1 | 0.2 ± 0.5 | 0.305 |
| Mitogen |  | 7.5 ± 1.9 | 7.9 ± 1.6 | 0.120 |
| MTB antigen - Nil |  | 0.1 ± 0.1 | 2.3 ± 2.1 | <0.001 |
| Cytokines (pg/ml) |  |  |  |  |
| IL-2 | 4.7 ± 2.2 | 5.7 ± 3.1 | 2.6 ± 1.8 | 0.610 |
| IL-10 | 21.2 ± 5.4 | 25.3 ± 8.1 | 12.9 ± 1 | 0.624 |
| TNF-alpha | 11.9 ± 1.7 | 13.6 ± 2.5 | 8.4 ± 0.8 | **0.038** |
| IL-22 | 33.7 ± 2.2 | 36.7 ± 3.2 | 27.7 ± 0.9 | 0.089 |
| IL-17A | 2.7 ± 0.8 | 3.5 ± 1.2 | 1.1 ± 0.5 | 0.065 |
| IL-17F | 460 ± 153 | 593 ± 227 | 190 ± 21.4 | **0.025** |
| IFN-gamma | 59.0 ± 30.8 | 80.8 ± 45.8 | 14.9 ± 6.4 | 0.482 |
| TGF-beta | 26.1 ± 1.0 | 26.4 ± 1.3 | 25.3 ± 1.5 | 0.647 |

Abbreviations: AST, aspartate transaminase; ALT, alanine transaminase; IL, interleukin; LTBI, latent tuberculosis infection; MTB, *Mycobacterium tuberculosis*; QFT, QuantiFERON-TB Gold In-Tube; TNF, tumor necrosis factor.

Data are presented as mean ± standard error of mean

P values were calculated using Student’s *t* test or the Mann–Whitney *U* test according to data normality.

**Additional file 1: Table S2**. Relative abundance (%) of the top 10 phyla in both the latent tuberculosis infection (LTBI) group and non-LTBI groups

| **Top 10 phyla** | **Non-LTBI** | **LTBI** | ***P*-value*** |
| --- | --- | --- | --- |
| *Bacteroidetes* | 47.949 | 51.962 | 0.481 |
| *Firmicutes* | 44.185 | 40.285 | 0.167 |
| *Proteobacteria* | 4.282 | 3.282 | 0.051 |
| *Actinobacteria* | 1.956 | 1.515 | 0.546 |
| *Fusobacteria* | 1.061 | 2.104 | 0.752 |
| *Verrucomicrobia* | 0.284 | 0.320 | 0.368 |
| *Cyanobacteria* | 0.051 | 0.339 | 0.539 |
| *Lentisphaerae* | 0.067 | 0.061 | 0.361 |
| *Synergistetes* | 0.038 | 0.059 | 0.327 |
| *Tenericutes* | 0.058 | 0.036 | 0.174 |

* According to a Mann–Whitney *U* test.

**Additional file 1: Table S3**. Relative abundance (%) of the top 10 genera in both latent tuberculosis infection (LTBI) group and non-LTBI group

| **Top 10 genera** | **Non-LTBI** | **LTBI** | ***p*-value*** |
| --- | --- | --- | --- |
| *Bacteroides* | 29.721 | 37.785 | 0.001 |
| *Prevotella_9* | 8.946 | 2.535 | 6.21E-06 |
| *Faecalibacterium* | 5.398 | 4.776 | 0.806 |
| *Alistipes* | 2.181 | 3.849 | 0.024 |
| *Parabacteroides* | 2.697 | 3.142 | 0.315 |
| *Blautia* | 2.237 | 3.000 | 0.013 |
| *Megamonas* | 2.550 | 1.956 | 0.093 |
| *Lactobacillus* | 2.929 | 1.535 | 0.222 |
| *Roseburia* | 2.105 | 2.222 | 0.994 |
| *Phascolarctobacterium* | 1.969 | 1.772 | 0.354 |

* According to a Mann–Whitney *U* test.

**Additional file 1: Table S4**. Relative abundance (%) of the top 10 genera only in the non-latent tuberculosis infection (non-LTBI) group

| **Top 10 genera** | **Non-LTBI** | **LTBI** | ***P*-value*** |
| --- | --- | --- | --- |
| *Rikenellaceae RC9 gut group* | 0.022 | 0 | 0.318 |
| *Sarcina* | 0.022 | 0 | 0.482 |
| *Allobaculum* | 0.009 | 0 | 0.318 |
| *Citrobacter* | 0.009 | 0 | 0.155 |
| *CHKCI002* | 0.007 | 0 | 0.079 |
| *Lachnospiraceae UCG-003* | 0.007 | 0 | 0.318 |
| *Acinetobacter* | 0.007 | 0 | 0.318 |
| *Atopobium* | 0.005 | 0 | 0.057 |
| *Calothrix PCC-6303* | 0.005 | 0 | 0.482 |
| *Pediococcus* | 0.005 | 0 | 0.318 |

* According to a Mann–Whitney *U* test.

**Additional file 1: Table S5**. Relative abundance (%) of the top 10 genera only in the latent tuberculosis infection (LTBI) group

| **Top 10 genera** | **Non-LTBI** | **LTBI** | ***P*-value*** |
| --- | --- | --- | --- |
| *Butyrivibrio* | 0 | 0.044 | 0.043 |
| *Clostridium sp. CAG:306* | 0 | 0.023 | 0.155 |
| *Candidatus Melainabacteria bacterium MEL.A1* | 0 | 0.008 | 0.155 |
| *Robinsoniella* | 0 | 0.006 | 0.155 |
| *UC5-1-2E3* | 0 | 0.004 | 0.155 |
| *Methanobrevibacter* | 0 | 0.002 | 0.155 |
| *Alloscardovia* | 0 | 0.002 | 0.155 |
| *Paraeggerthella* | 0 | 0.002 | 0.155 |
| *Clostridium sensu stricto 13* | 0 | 0.002 | 0.155 |
| *Murdochiella* | 0 | 0.002 | 0.155 |

* According to a Mann–Whitney *U* test.

**Additional file 1: Table S6.** Relative abundance (%) of the 26 most differential genera between the latent tuberculosis infection (LTBI) and non-LTBI groups

| **Genus** | **Non-LTBI** | **LTBI** | ***P*-value*** |
| --- | --- | --- | --- |
| *Prevotella 9* | 8.946 | 2.535 | 6.21E-06 |
| *Bacteroides* | 29.721 | 37.785 | 1.31E-03 |
| *Actinomyces* | 0.043 | 0.004 | 2.27E-03 |
| *Eisenbergiella* | 0.020 | 0.046 | 3.06E-03 |
| *Haemophilus* | 0.331 | 0.025 | 3.33E-03 |
| *Intestinimonas* | 0.072 | 0.054 | 4.02E-03 |
| *Muribaculaceae metagenome* | 0.315 | 0.038 | 5.47E-03 |
| *Rothia* | 0.014 | 0.002 | 6.46E-03 |
| *Hydrogenoanaerobacterium* | 0.002 | 0.008 | 9.28E-03 |
| *Candidatus Soleaferrea* | 0.020 | 0.015 | 1.14E-02 |
| *Uncultured Muribaculaceae* | 0 | 0.153 | 1.30E-02 |
| *Blautia* | 2.237 | 3.000 | 1.33E-02 |
| *Streptococcus* | 2.614 | 0.956 | 2.10E-02 |
| *Ruminococcaceae UCG-013* | 0.036 | 0.040 | 2.21E-02 |
| *Alistipes* | 2.181 | 3.849 | 2.41E-02 |
| *[Eubacterium] xylanophilum group* | 0.007 | 0.048 | 2.56E-02 |
| *Ruminiclostridium 6* | 0.116 | 0.715 | 3.04E-02 |
| *Lachnoclostridium* | 1.014 | 1.443 | 3.44E-02 |
| *Uncultured Rhodospirillales* | 0.025 | 0.293 | 3.80E-02 |
| *Anaerotruncus* | 0.022 | 0.056 | 4.08E-02 |
| *Butyrivibrio* | 0 | 0.044 | 4.34E-02 |
| *Uncultured Clostridiales Family XIII* | 0 | 0.002 | 4.34E-02 |
| *Proteus* | 0 | 0.002 | 4.34E-02 |
| *Flavonifractor* | 0.197 | 0.289 | 4.71E-02 |
| *Butyricicoccus* | 0.246 | 0.312 | 4.97E-02 |
| *Acidaminococcus* | 0.458 | 0.303 | 4.99E-02 |

* According to a Mann–Whitney *U* test.

**Additional file 1: Table S7.** Performance of predictive models that included different numbers of the 26 most differential genera between the latent tuberculosis infection (LTBI) and non-LTBI groups

| **No. of genera used** | **AUROC** | **Accuracy** | **Sensitivity** | **Specificity** | **F1 score*** |
| --- | --- | --- | --- | --- | --- |
| 2 | 0.641 | 0.641 | 0.462 | 0.731 | 0.462 |
| 3 | 0.741 | 0.718 | 0.385 | 0.885 | 0.476 |
| 4 | 0.820 | 0.821 | 0.615 | 0.923 | 0.696 |
| 5 | 0.811 | 0.846 | 0.692 | 0.923 | 0.750 |
| 6 | 0.834 | 0.872 | 0.769 | 0.923 | 0.800 |
| 7 | 0.891 | 0.795 | 0.538 | 0.923 | 0.636 |
| 8 | 0.896 | 0.769 | 0.538 | 0.885 | 0.609 |
| 9 | 0.917 | 0.846 | 0.692 | 0.923 | 0.750 |
| 10 | 0.926 | 0.846 | 0.692 | 0.923 | 0.750 |
| 11 | 0.929 | 0.821 | 0.615 | 0.923 | 0.696 |
| 12 | 0.929 | 0.821 | 0.615 | 0.923 | 0.696 |
| 13 | 0.926 | 0.821 | 0.615 | 0.923 | 0.696 |
| 14 | 0.935 | 0.821 | 0.615 | 0.923 | 0.696 |
| 15 | 0.933 | 0.821 | 0.615 | 0.923 | 0.696 |
| 16 | 0.923 | 0.795 | 0.538 | 0.923 | 0.636 |
| 17 | 0.926 | 0.795 | 0.538 | 0.923 | 0.636 |
| 18 | 0.923 | 0.846 | 0.692 | 0.923 | 0.750 |
| 19 | 0.843 | 0.744 | 0.462 | 0.885 | 0.545 |
| 20 | 0.864 | 0.744 | 0.462 | 0.885 | 0.545 |
| 21 | 0.849 | 0.744 | 0.462 | 0.885 | 0.545 |
| 22 | 0.837 | 0.744 | 0.462 | 0.885 | 0.545 |
| 23 | 0.849 | 0.769 | 0.462 | 0.923 | 0.571 |
| 24 | 0.883 | 0.769 | 0.462 | 0.923 | 0.571 |
| 25 | 0.845 | 0.795 | 0.615 | 0.885 | 0.667 |
| 26 | 0.870 | 0.795 | 0.615 | 0.885 | 0.667 |

Abbreviation: AUROC, areas under the receiver operating characteristic curve

*The F1 score is the harmonic average of precision and recall, calculated using the formula: $F1=(2\times Precision\times Recall)/(Precision+Recall)$.

**Additional file 1: Table S8.** Confusion matrix of the classifier involving 6 genera and a test set (39 samples [30%]) for differentiating between latent tuberculosis infection (LTBI) and non-LTBI groups, as determined by a random forest model

| Class | | Actual | |
| --- | --- | --- | --- |
|  |  | **Positive** | **Negative** |
| Prediction | Positive | 10 | 2 |
|  | Negative | 3 | 24 |

**Additional file 1: Table S9.** P values for each of the 6 selected genera in linear regression models for predicting the plasma levels of individual cytokines and the proposed model performance

|  | IFN-γ | IL-17A | IL-17F | IL-2 | IL-10 | IL-22 | TGF-β | TNF-α |
| --- | --- | --- | --- | --- | --- | --- | --- | --- |
| *Bacteroides* | **0.050** | **0.019** | 0.259 | **0.088** | **0.036** | **0.011** | 0.304 | **0.025** |
| *Prevotella_9* | 0.257 | **0.074** | 0.433 | 0.245 | 0.303 | **0.025** | 0.491 | 0.206 |
| *Streptococcus* | 0.818 | 0.357 | 0.853 | 0.771 | 0.648 | 0.519 | 0.718 | 0.996 |
| *Alistipes* | 0.156 | 0.244 | 0.242 | 0.324 | 0.193 | 0.119 | 0.158 | 0.297 |
| *Actinomyces* | 0.400 | 0.365 | 0.478 | 0.384 | 0.495 | 0.344 | 0.616 | 0.469 |
| *Blautia* | **0.0005** | **0.004** | **0.001** | **0.003** | **0.000** | **0.030** | 0.147 | **0.001** |
|  |  |  |  |  |  |  |  |  |
| Model performance |  |  |  |  |  |  |  |  |
| *R*^2^ | 0.134 | 0.122 | 0.110 | 0.105 | 0.150 | 0.108 | 0.040 | 0.129 |
| p value | **0.006** | **0.012** | **0.024** | **0.031** | **0.002** | **0.026** | 0.524 | **0.009** |

Abbreviations: IFN, interferon; IL, interleukin; TGF, transforming growth factor;

TNF, tumor necrosis factor

**Additional file 1: Table S10.** Change of model performance for predicting the plasma levels of individual cytokines before and after including the 6 selected genera into the linear regression models containing 11 potential confounders (sex, body-mass index ≥27 kg/m^2^, and use of metformin, DDP4 inhibitor, SGLT2 inhibitor, sulfonylurea, thiazolidinedione, meglitinides, acarbose, and use of ≥3 oral antidiabetic drugs, as well as statin)

|  | IFNγ | | IL17A | | IL17F | | IL2 | | IL10 | | IL22 | | TGFβ1 | | TNFα | |
| --- | --- | --- | --- | --- | --- | --- | --- | --- | --- | --- | --- | --- | --- | --- | --- | --- |
|  | *R^2^* | *p* | *R^2^* | *p* | *R^2^* | *p* | *R^2^* | *p* | *R^2^* | *p* | *R^2^* | *p* | *R^2^* | *p* | *R^2^* | *p* |
| After adding 6 genera | 0.209 | 0.045 | 0.183 | 0.116 | 0.192 | 0.087 | 0.188 | 0.098 | 0.204 | 0.054 | 0.173 | 0.16 | 0.132 | 0.466 | 0.185 | 0.108 |
| Before adding 6 genera | 0.080 | 0.508 | 0.065 | 0.698 | 0.103 | 0.277 | 0.092 | 0.375 | 0.064 | 0.704 | 0.048 | 0.869 | 0.098 | 0.316 | 0.059 | 0.766 |

Abbreviations: IFN, interferon; IL, interleukin; TGF, transforming growth factor; TNF, tumor necrosis factor

**Additional file 1: Figure S1**. Rarefaction curve of sequencing data from 130 fecal samples. Samples with fewer than 45,000 sequences were excluded, and the remaining samples were rarefied to 49,423 sequences (range: 32,136 to 121,226) per sample for subsequent ordinations and permutational multivariate ANOVA analysis.

Abbreviations: ASVs, amplicon sequence variants

**Additional file 1: Figure S2. Biodiversity and *Prevotella/Bacteroides* (P/B) ratio of gut microbial communities in diabetes mellitus (DM) patients with or without latent tuberculosis infection (LTBI). (A)** Beta diversity was determined by a principal coordinates analysis plot based on the unweighted UniFrac distance. Each dot represents one sample. **(B)** *Prevotella/Bacteroides* (P/B) ratio between the latent tuberculosis infection (LTBI) and non-LTBI groups. *** represents a P value of <0.001 according to a Mann–Whitney *U* test.

**Additional file 1: Figure S3 Biodiversity of gut microbial communities in diabetes mellitus (DM) patients with or without potential confounders:** (A) sex, (B) BMI ≥ 27kg/m^2^, (C) Metformin, (D) DDP4 inhibitor, (E) SGLT2 inhibitor, (F) Sulfonylurea, (G) Thiazolidinedione, (H) Meglitinide, (I) Acarbose, (J) Statin


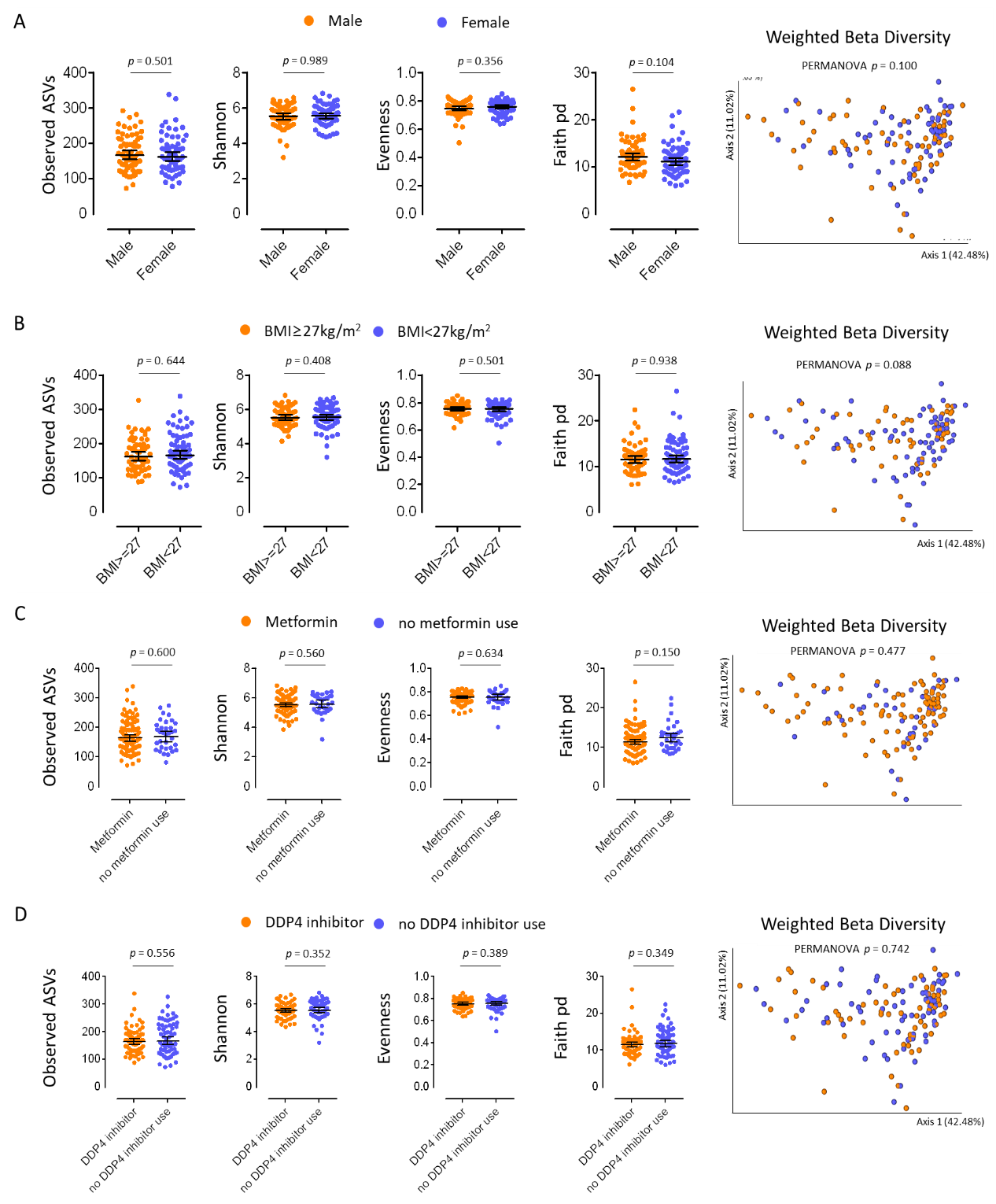


**
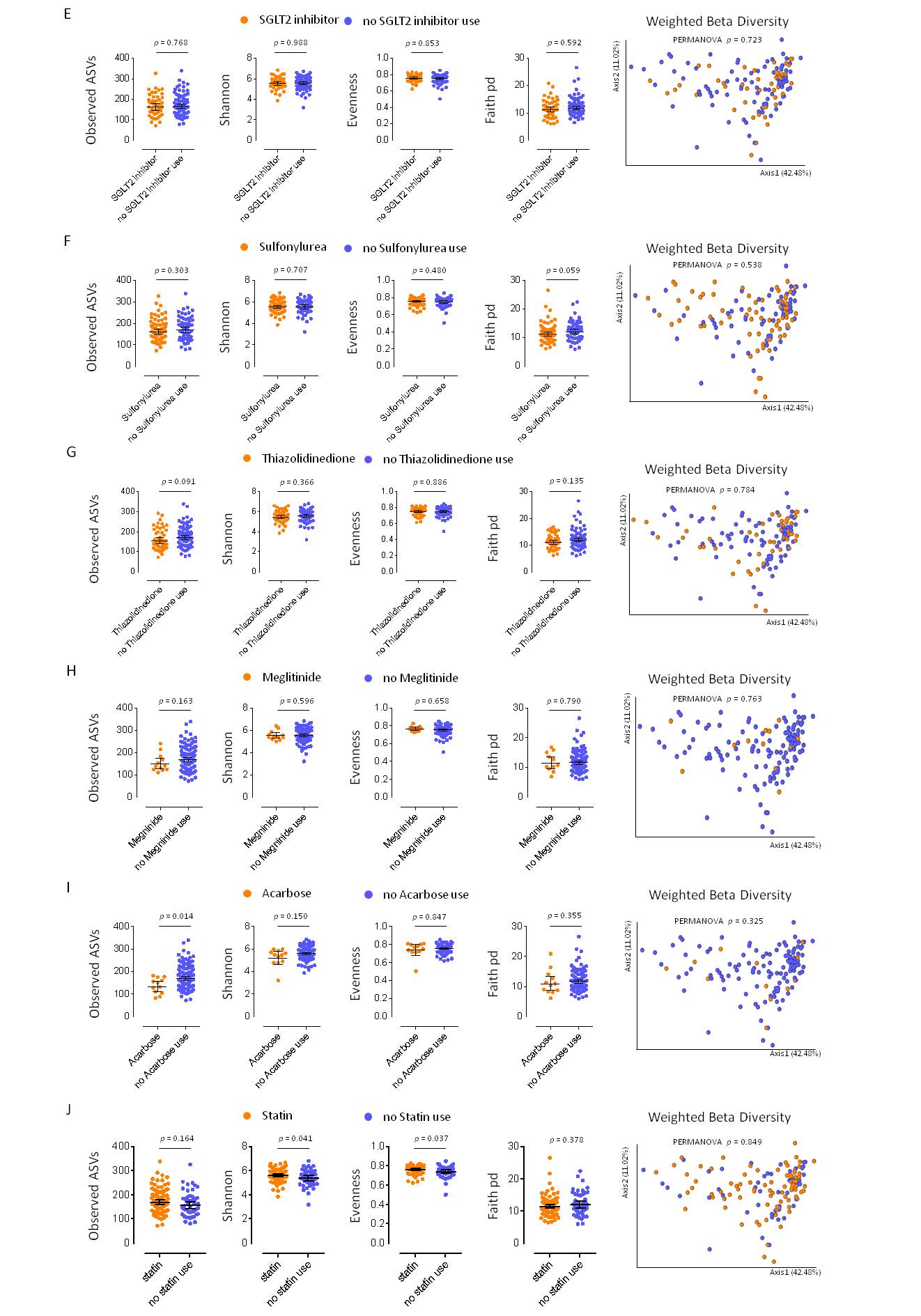
**

**Additional file 1: Figure S4.** The feature importance of the 6 selected genera and potential confounders in the predicting models for serum cytokine levels


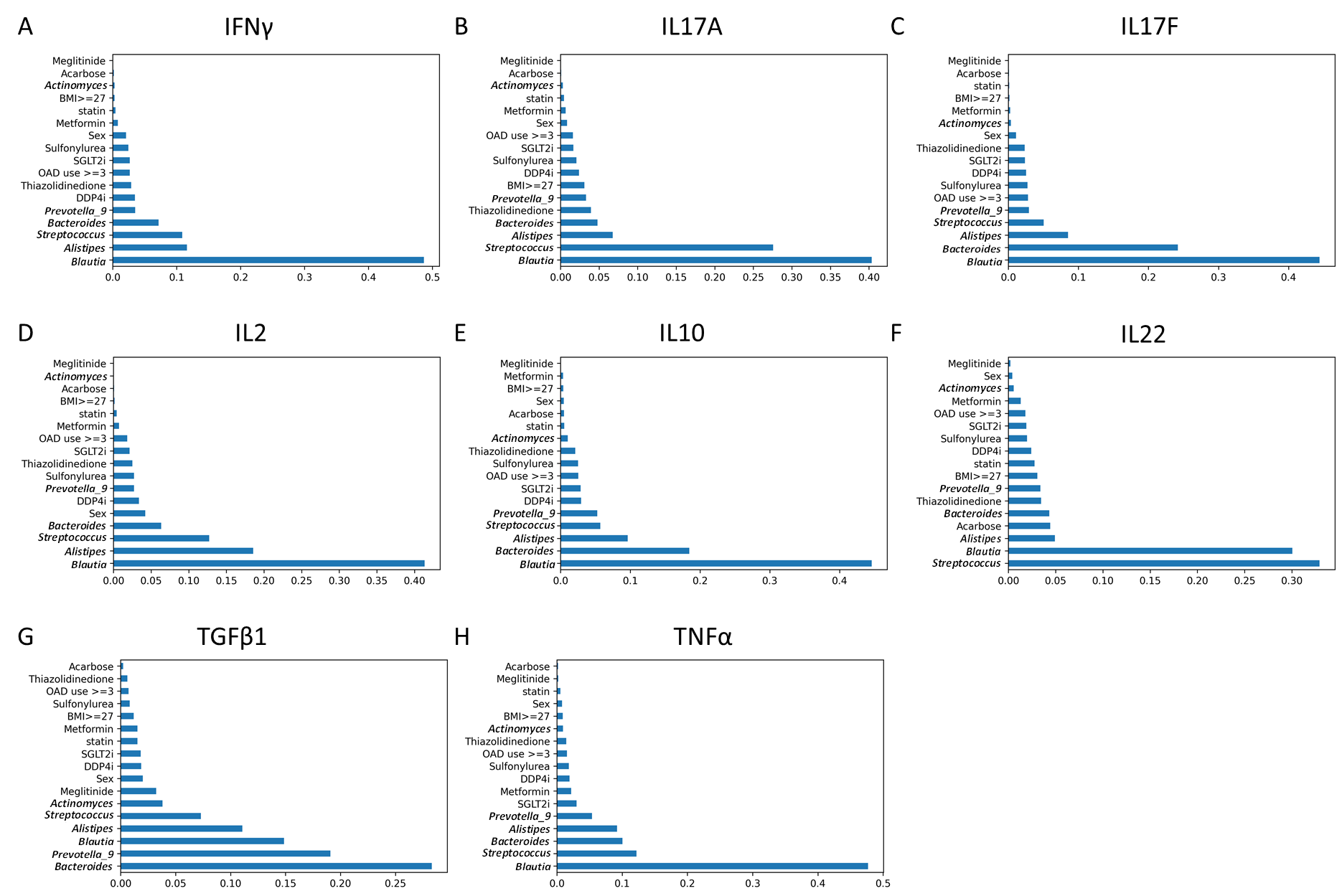


Abbreviations: BMI: body-mass index (kg/m^2^); OAD, oral antidiabetic drugs; DDP4i, Dipeptidyl peptidase 4 inhibitor;
SGLT2i, sodium-glucose transport protein 2 inhibitor

**Additional file 1: Figure S5**. Prediction of gene function of the 6 most differential genera between latent tuberculosis infection (LTBI) and non-LTBI groups using PICRUSt2.


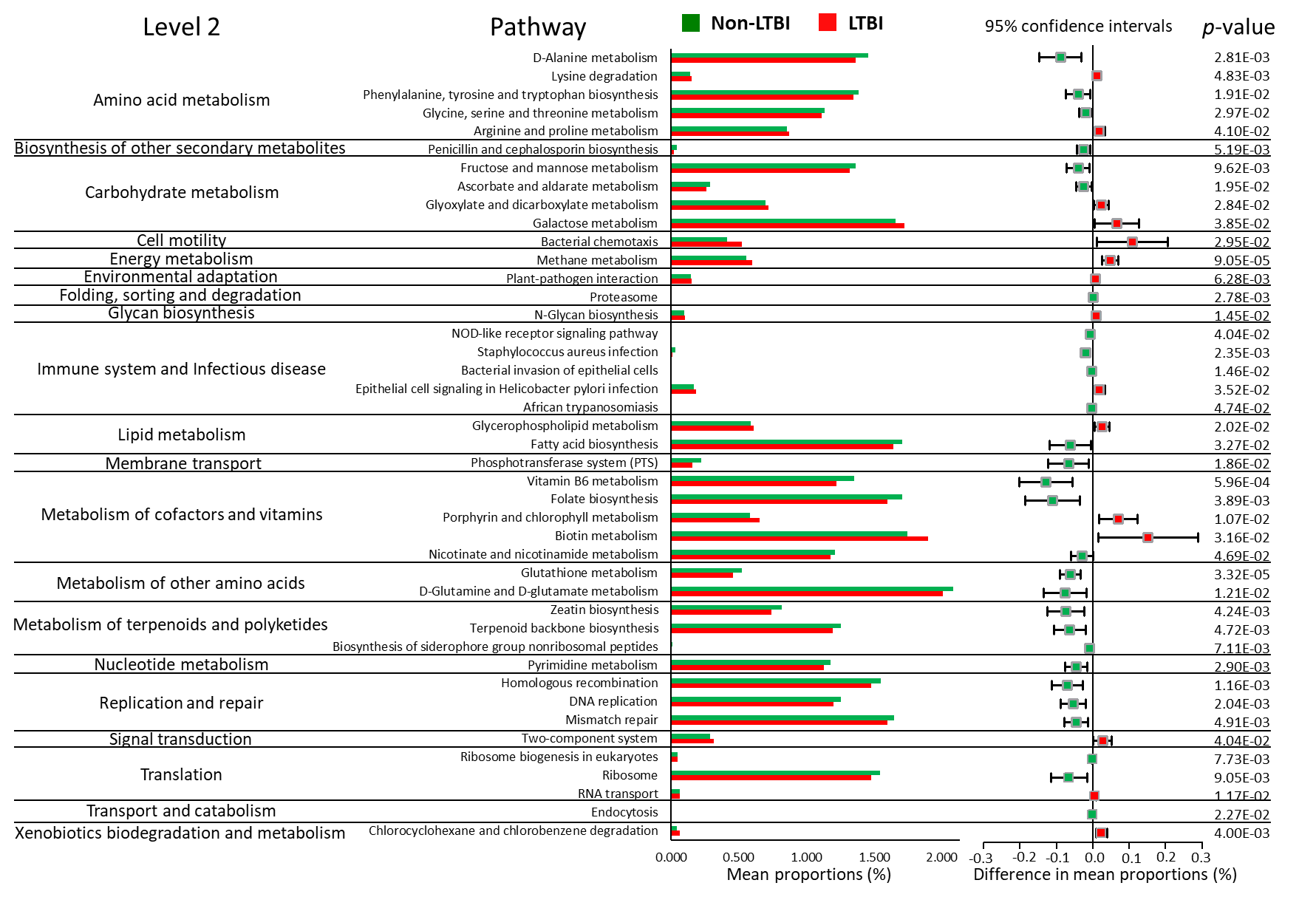

Supplement: Supplementary file 1 — Additional file 1: Table S1. Laboratory and cytokine analysis results for the 130 patients with poorly controlled diabetes mellitus. Table S2. Relative abundance (%) of the top 10 phyla in both the latent tuberculosis infection (LTBI) group and non-LTBI groups. Table S3. Relative abundance (%) of the top 10 genera in both latent tuberculosis infection (LTBI) group and non-LTBI group. Table S4. Relative abundance (%) of the top 10 genera only in the non-latent tuberculosis infection (non-LTBI) group. Table S5. Relative abundance (%) of the top 10 genera only in the latent tuberculosis infection (LTBI) group. Table S6. Relative abundance (%) of the 26 most differential genera between the latent tuberculosis infection (LTBI) and non-LTBI groups. Table S7. Performance of predictive models that included different numbers of the 26 most differential genera between the latent tuberculosis infection (LTBI) and non-LTBI groups. Table S8. Confusion matrix of the classifier involving 6 genera and a test set (39 samples [30%]) for differentiating between latent tuberculosis infection (LTBI) and non-LTBI groups, as determined by a random forest model. Table S9. P values for each of the 6 selected genera in linear regression models for predicting the plasma levels of individual cytokines and the proposed model performance. Table S10. Change of model performance for predicting the plasma levels of individual cytokines before and after including the 6 selected genera into the linear regression models containing 11 potential confounders (sex, body-mass index ≥ 27 kg/m2, and use of metformin, DDP4 inhibitor, SGLT2 inhibitor, sulfonylurea, thiazolidinedione, meglitinides, acarbose, and use of ≥ 3 oral antidiabetic drugs, as well as statin). Figure S1. Rarefaction curve of sequencing data from 130 fecal samples. Samples with fewer than 45,000 sequences were excluded, and the remaining samples were rarefied to 49,423 sequences (range: 32,136 to 121,226) per sample for subsequent ordinat [file 12931_2023_2312_MOESM1_ESM.docx]
